# The role of endoplasmic reticulum stress in maintaining and targeting multiple myeloma: a double-edged sword of adaptation and apoptosis

**DOI:** 10.3389/fgene.2013.00109

**Published:** 2013-06-11

**Authors:** Shai White-Gilbertson, Yunpeng Hua, Bei Liu

**Affiliations:** Department of Microbiology and Immunology, Hollings Cancer Center, Medical University of South CarolinaCharleston, SC, USA

**Keywords:** endoplasmic reticulum, unfolded protein response, multiple myeloma, ER stress, apoptosis

## Abstract

Increased cellular protein production places stress on the endoplasmic reticulum (ER), because many of the nascent proteins pass through the ER for folding and trafficking. Accumulation of misfolded proteins in the ER triggers the activation of three well-known pathways including IRE1 (inositol requiring kinase 1), ATF6 (activating transcription factor 6), and PERK (double stranded RNA-activated protein kinase-like ER kinase). The activity of each sensor modulates the overall ER strategy for managing protein quality control as cellular needs change due to growth, differentiation, infection, transformation, and host of other possible physiological states. Here we review the role of ER stress in multiple myeloma (MM), an incurable plasma cell neoplasm. MM is closely linked to dysregulated unfolded protein response in the ER due to the heightened production of immunoglobulin and the metabolic demands of malignant uncontrolled proliferation. Together, these forces may mean that myeloma cells have an “Achilles heel” which can be exploited as a treatment target: their ER stress response must be constitutively active at a remarkably high level to survive their unique metabolic needs. Therefore, inhibition of the ER stress response is likely to injure the cells, as is any further demand on an already over-worked system. Evidence for this vulnerability is summarized here, along with an overview of how each of the three ER stress sensors has been implicated in myeloma pathogenesis and treatment.

## INTRODUCTION

Multiple myeloma (MM) is a cancer of plasma cells, the antibody-producing end stage of B cell development. Plasma cells are the originating cell for a variety of diseases, collectively known as plasma cell dyscrasias, including systemic light-chain amyloidosis, monoclonal gammopathy of undermined significance (MGUS), solitary plasmacytoma, smoldering myeloma, and MM. In each case, the hallmark of the pathology is the overproduction of a secreted protein by a diseased plasma cell population (Barlogie et al., 1992). In this review, we will focus on MM, a cancer with over 20,000 new diagnoses expected in 2013 by the National Cancer Institute in the United States (http://www.cancer.gov/cancertopics/types/myeloma/). MM typically presents as an incurable disease, almost inevitably recurring after therapy (Munshi and Anderson, 2013). Nonetheless, the introduction of the proteasome inhibitor bortezomib to treatment regimens represented a breakthrough for myeloma patients by increasing survival time significantly (Moreau, 2012). The sensitivity of myeloma cells to bortezomib may be due in part to the specialized metabolism of plasma cells, which are adapted to generate large volumes of secreted immunoglobulins and operate with an elevated baseline demand on the endoplasmic reticulum (ER). This may be a liability for myeloma cells, which are additionally burdened with the protein production necessary for malignant proliferation. The resulting vulnerability to further perturbation in protein metabolism may offer a partial explanation for the success of bortezomib (Landowski et al., 2005; Obeng et al., 2006; Meister et al., 2007). Efforts to understand and target the integrated ER stress response in myeloma will be summarized here, with a focus on the three ER stress sensors that coordinate this response: inositol requiring kinase 1 (IRE1; Sidrauski and Walter, 1997; Yoshida et al., 2001), double stranded RNA-activated protein kinase-like ER kinase (PERK; Harding et al., 2000), and the transcription factor activating transcription factor 6 (ATF6; Yoshida et al., 2000). Each of these sensors is located at the apex of a pathway, and each is capable of inducing the expression of several major ER heat shock proteins and enhancing protein folding machinery (Malhotra and Kaufman, 2007).

All three ER stress response sensors are embedded in the ER membrane where they are normally bound by the ER chaperone grp78 (alias BiP; Ma et al., 2002; Sommer and Jarosch, 2002; Kimata et al., 2004). This binding inhibits the activity of each sensor. Grp78 releases the sensors in response to mounting ER stress as its chaperone functions are required (Lee, 2005). However, this is not a uniform method of control over the three combined sensors; different cellular conditions result in differing patterns of sensor activation. For example, during B cell differentiation only two sensors, IRE1 and ATF6 are activated while the third, PERK, is not (Ma et al., 2010). Using a B cell line capable of induction of all three ER stress sensors and capable of differentiation into plasma cells, Ma et al. (2010) demonstrated that IRE1 is activated quickly upon exposure to differentiation-inducing lipopolysaccharide (LPS) treatment, with ATF6 activation following secondarily. In contrast, PERK activation could not be elicited from these cells upon differentiation, even when treated with the ER stressor thapsigargin, although this treatment could stimulate PERK activity before differentiation (Ma et al., 2010).

**FIGURE 1:**
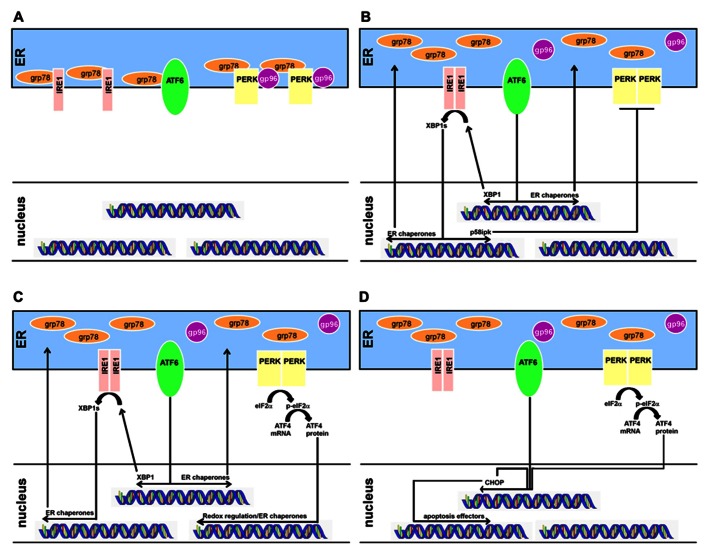
**The progression of ER stress responses in multiple myeloma development and treatment.** **(A)** Schematic of B-cell ER stress activity before differentiation. The ER stress sensors IRE1, ATF6, and PERK are bound to ER chaperones which inhibit either homodimerization (for IRE1 and PERK) or translocation to the nucleus (ATF6). Transcriptomes downstream of the ER sensors are inactive. **(B)** Upon differentiation into a plasma cell, protein production is increased in a regulated fashion. Chaperones release the ER stress sensors but PERK remains inhibited through its IRE1-controlled inhibitor p58ipk. IRE1 and ATF4 upregulate ER chaperones to assist with the protein production involved in secreting antibody. **(C)** Myeloma cells further activate the ER stress programs, involving PERK activation. All three arms are used by myeloma cells, although the CHOP response is limited. **(D)** Myeloma cells can be induced to further increase their ER stress response, tipping the system towards cell death. When ATF4 and ATF6 coordinate to induce transcription of CHOP, a pro-apoptosis transcription factor, the ER stress response moves from adaptive to destructive. This is the therapeutic goal of some drug regimens, such as bortezomib.

Crosstalk between the sensor systems provides additional control over the cellular response. For example, one effect of IRE1 activation is the transcription of a PERK inhibitor named p58ipk (Iwakoshi et al., 2003; Ma et al., 2010). In addition, ATF6 and PERK appear to converge on signaling through the transcription factor CHOP (C/EBP homologous protein; Okada et al., 2002). Thus, both re-enforcement and antagonism exist between the sensors, allowing a highly tunable response based on cellular needs (**Figure 1**).

Due to the baseline ER stress present in untransformed plasma cells, myeloma is a particularly complex disease in which to examine ER stress. Several excellent reviews have addressed ER stress response more generally (Woehlbier and Hetz, 2011; Logue et al., 2013; Schonthal, 2013), so here we will provide brief overviews of the components and focus on experimental data which elucidates their role in myeloma disease, including responsiveness to chemotherapeutics.

## IRE1

### OVERVIEW OF IRE1 FUNCTION

Inositol requiring kinase 1 is a bifunctional transmembrane kinase and endoribonuclease. It was first identified in yeast, called Ire1p, which is correlated with unfolded protein response (UPR; Cox et al., 1993). Upon activation of the UPR, Ire1p oligomerizes, phosphorylates, and initiates splicing of homologous to ATF/CREB1 (HAC1; Shamu and Walter, 1996; Sidrauski and Walter, 1997). IRE1 is conserved in all eukaryotic cells. In mammalian cells, there are two forms of IRE1, IRE1α, and IRE1β. Most cells and tissues express IRE1α, while only intestinal epithelial cells express IRE1β. IRE1α and IRE1β have similar cleavage specificities (Tirasophon et al., 1998; Wang et al., 1998; Patil and Walter, 2001). Previous studies have demonstrated that *X box binding protein 1 (XBP1)* mRNA is a substrate for the endoribonuclease activity of IRE1. Upon activation of the UPR, the IRE1 RNase activity initiates and removes a 26 nucleotide intron from *XBP1* mRNA (Yoshida et al., 2001; Calfon et al., 2002; Lee et al., 2002). This splicing form of XBP1, denoted XBP1s, is a transcriptional activator that plays an important role in activation of a variety of UPR target genes, which include ERdj4, p58^IPK^, DnaJ/Hsp40-like genes, ER degradation enhancer, mannosidase alpha-like (EDEM), human ER-associated DNAJ (HEDJ), protein disulfide isomerase-P5 (PDI-P5), and ribosome-associated membrane protein 4 (RAMP4; Lee et al., 2003).

### IRE1/XBP1 PATHWAY IS ESSENTIAL FOR PLASMA CELL DIFFERENTIATION

Both IRE1 and XBP1 are critical for plasma cell differentiation. Genetic deletion of XBP1 causes lack of plasma cells, with concomitantly decreased baseline and antigen specific serum level of immunoglobulin (Reimold et al., 2001; Iwakoshi et al., 2003; Shaffer et al., 2004). In addition, IRE1α is required to splice XBP1 for terminal differentiation of mature B cells into antibody-secreting plasma cells as demonstrated by using an IRE1α-deficient chimeric mouse model (Zhang et al., 2005). Furthermore, in IRE1α conditional knockout mice, the serum levels of IgM and IgG1 are reduced by half compared with the control mice. However, the IgM^+^, IgD^+^, and B220^+^ populations are similar between IRE1α conditional knockout mice and control mice. This result suggests that IRE1α is required for efficient plasma cell production of antibodies, and is critical for final B cell differentiation into a plasma cell (Iwawaki et al., 2010). These studies suggest that the IRE1/XBP1 pathway is required for differentiation and survival of cell types that secrete high levels of protein.

### IRE1/XBP1 IS POTENTIAL THERAPEUTIC TARGET FOR MULTIPLE MYELOMA

In addition to the critical roles of IRE1/XBP1 in plasma cell differentiation, a picture has emerged for the roles of UPR in myeloma. Indeed, XBP1s and downstream ER chaperones are consistently up regulated in myeloma patients. Patients with a low XBP1 spliced/unspliced ratio (≤1.33) have a longer overall survival compared with those with a higher ratio (*p* = 0.03, median, 56 vs 40 months; HR = 1.75; 95% CI = 1.07–2.85; Bagratuni et al., 2010). Moreover, transgenic expression of XBP1s in mice also leads to plasma cell dyscrasia with evidence of increased monoclonal antibodies (“M-spike”), lytic bone lesions, plasmacytosis, and kidney damage (Carrasco et al., 2007). Given this information, IRE1/XBP1 could be a potential therapeutic target for MM.

To investigate whether blocking the IRE1/XBP1 pathway is a therapeutic for MM, researchers performed chemical library screening and they identified a small-molecule, STF-083010, that specifically blocks the endonuclease activity of IRE1 without affecting its kinase activity (Papandreou et al., 2011). Furthermore, they treated different myeloma cell lines with different doses of STF-083010 *in vitro* and demonstrated that this compound causes myeloma cell death. Importantly, STF-083010 is also selectively cytotoxic to freshly isolated CD138^+^ plasma cells from myeloma patients compared with CD19^+^ B cells, CD3^+^ T cells, and CD56^+^ NK (natural killer) cells. Finally, treatment of human myeloma xenografts in NSG (NOD scid gamma) mice was performed. STF-083010 was given by intraperitoneal injection on day 1 and day 8 and this compound significantly inhibited the growth of these tumors *in vivo* (Papandreou et al., 2011). In addition, another small-molecule, MKC-3946, also blocks the IER1α endoribonuclease domain. MKC-3946 inhibits multiple human myeloma cell lines without toxicity to normal mononuclear cells. MKC-3946 also blocks ER stress induced by both bortezomib and heat shock protein 90 inhibitor 17-AAG. In addition, MKC-3946 significantly enhanced cytotoxicity induced by bortezomib or 17-AAG (Mimura et al., 2012). A similar result was found by using an XBP1 inhibitor, toyocamycin, which was identified from the culture broth of an Actinomycete strain. Toyocamycin has been shown to suppress the XBP1 mRNA splicing in HeLa cells which is induced by thapsigargin, tunicamycin, and 2-deoxyglucose. It does not, however, affect ATF6 and PERK activation. Although toyocamycin does not inhibit IRE1α phosphorylation, it prevents IRE1α-induced XBP1 mRNA cleavage and inhibits constitutive activation of XBP1 expression in myeloma cell lines as well as in samples from myeloma patients *in vitro*. Toyocamycin also induces apoptosis of myeloma cells, including bortezomib-resistant myeloma cells *in vitro*, and it also inhibits myeloma cell growth in a human myeloma xenograft model (Ri et al., 2012). Taken together, these results demonstrate that blockade of IRE1/XBP1 pathway by small-molecule compounds is a potential therapeutic for treatment of human myeloma.

## ATF6

### OVERVIEW OF ATF6 FUNCTION

Among the three ER stress sensors, only ATF6 does not dimerize to potentiate enzymatic activity. Instead, under ER stress conditions, ATF6 translocates to the Golgi apparatus and it is processed by site 1 protease (S1P) and site 2 protease (S2P) to release an active form of ATF6 (ATF6f). ATF6f translocates to the nucleus and activates target genes (Chen et al., 2002). In this capacity, ATF6 works in partnership with IRE1, as one of the target genes of ATF6 is XBP1, the key substrate of IRE1 (Yoshida et al., 2001). In addition to fueling the IRE1 arm of the ER stress response, ATF6 also functions as a transcription factor for ER chaperone proteins, thereby easing ER burden (Arai et al., 2006). These contributions to the ER stress response complement IRE1 activation and are generally adaptive, allowing such upregulation of protein production as is seen in plasma cell development. However, prolonged ATF6 activation can also result in transcription of CHOP, another transcription factor which enacts a largely apoptotic program of gene expression (Matsumoto et al., 1996). This effect of ATF6 activity occurs in conjunction with PERK activation, in contrast to the protective program that ATF6 and IRE1 jointly support.

One group has made an attempt in HeLa cells to describe the genetic modulation downstream of ATF6 activation and to distinguish it from the genetic signature of PERK activation (Okada et al., 2002). The group examined this question by comparing the cellular pool of mRNA in HeLa cells treated with the general ER stress inducer tunicamycin with that of cells stably expressing the nuclear form of ATF6. From this experiment, the ATF6 contribution to the integrated ER stress response was extracted for HeLa cells. The primary targets identified were the expected ER chaperones grp78, gp96, and calreticulin (Okada et al., 2002). In addition, proteins which directly modify disulphide bonds to assure proper folding of nascent proteins were identified, such as ERp62 and ERp71 (Okada et al., 2002). Unfortunately, the authors concluded that this cell system was not conducive to the study of XBP1 transcription, which is critical for understanding myeloma development and progression. However, the research revealed that ATF6 and PERK both converge on CHOP transcription, confirming this as a locus of crosstalk between the two sensors (Okada et al., 2002).

CHOP (C/EBP homologous protein, alias GADD153) is a pro-apoptotic transcription factor routinely used as a read-out for activation of the ER stress response (Kawabata et al., 2012; Mimura et al., 2012; Schonthal, 2013). The Mori group has proposed that CHOP transcription is most efficiently activated upon binding by both the nuclear form of ATF6 and ATF4, the transcription factor effector of PERK activation (Okada et al., 2002). The convergence of ER stress signals results in CHOP binding to its target genes, with inhibitory effects on some targets and transcriptional effects on others. CHOP activity results in the downregulation of the anti-apoptotic Bcl2 (B-cell lymphoma 2) as well as the upregulation of the ER-resident oxidase ERO1-alpha (Marciniak et al., 2004). CHOP is also its own target, suggesting that its activation constitutes a commitment to programmed cell death (Marciniak et al., 2004).

### ATF6 IN MULTIPLE MYELOMA

Surprisingly little has been written about the role of ATF6 in MM, especially considering the important role it plays in the generation of the IRE1 substrate XBP1 (Lee et al., 2002). Indeed, the transcriptome of ATF6 should itself be a discrete target of research in the myeloma field.

One group has performed specific knockdown of ATF6 in myeloma cells and shown that, as is also the case for the other ER stress sensors, targeted loss resulted in significant cell death (Michallet et al., 2011). In addition, increased baseline signaling through the PERK sensor was enhanced upon knockdown of ATF6. Thus, the three sensors appear to all be required for baseline survival for myeloma cells, although crosstalk may allow for some limited compensation between the sensor systems.

Certainly, the crosstalk between ATF6 and the other two ER stress sensors suggests that ATF6 plays the role of a “swing vote.” When activated in conjunction with IRE1, growth and adaptation to protein production is reinforced. When linked to PERK, ATF6 activity can support a programmed cell death response. This duality indicates a potentially powerful target, identifying ATF6 as an understudied aspect of myeloma.

## PERK

### OVERVIEW OF PERK FUNCTION

The pancreatic eIF2-alpha kinase (PERK, alias EIF2αK3) is the third known sensor of ER stress and like the other two, it is embedded in the ER membrane. As the only such sensor left inactivated in the normal development of plasma cells, it has been of particular interest in the study of myeloma (Ma et al., 2010). We will therefore provide a summary of its canonical function and then review studies testing the role of PERK in baseline myeloma biology and in response to drug treatment.

Like the other two ER stress sensors, the activation of PERK requires its release by grp78. In addition, the chaperone gp96 (alias grp94) has been shown to bind PERK at baseline and release it during ER stress conditions (Ma et al., 2002). Upon release, PERK is free to homodimerize and activate as a kinase. Active PERK has three interacting mechanisms, allowing gradations of cellular effects ranging from protective to destructive. These effects are mediated by eIF2-alpha, ATF4, and CHOP. First, the direct phosphorylation target of PERK is eIF2-alpha, a protein needed for ribosomal translation of mRNA (Wek and Cavener, 2007). The phosphorylation of eIF2-alpha inhibits its activity, resulting in global repression of protein production. This strategy of translation repression reduces the load of nascent proteins being delivered to the ER for processing and is an effective short-term answer to the problem of ER stress. However, the side effects of halting protein production are myriad, and the phosphorylation of eIF2-alpha does allow exceptions. For instance, mRNA with IRES (internal ribosome entry site) sequences can still be translated under these conditions (Gerlitz et al., 2002). In addition, the transcription factor ATF4 is translated and subsequently translocates to the nucleus. The mechanism allowing such translation during eIF2α phosphorylation has been of significant interest and research has identified a double upstream open reading frame structure in the ATF4 mRNA which is preferentially translated when ribosomal processing is slowed (Lu et al., 2004; Kilberg et al., 2009). ATF4 then binds to genetic sequences with CCAAT (cytidine-cytidine-adenosine-adenosine-thymidine) motifs, many of which can be translated under the phosphorylated eIF2a condition which is downstream of PERK activation, likely due to upstream ORFs (open reading frames) that function like the ones present in ATF4 mRNA (Lu et al., 2004; Kilberg et al., 2009). This activation of the ATF4 transcriptome is the second major arm of PERK response to ER stress.

ATF4 facilitates the transcription mRNAs coding for proteins with functions specific to ER stress conditions. For instance, redox-management genes are turned on, as well as additional chaperones for the ER (Harding et al., 2003; Liu et al., 2008; Ye and Koumenis, 2009). Again, this strategy is adaptive for the cell and may allow the cell to cope with short-term challenges. However, the third arm of PERK signaling involves activation of CHOP, already described as a target of ATF6. The CHOP promoter includes binding sites for both ATF4 and ATF6, which appear to synergize (Okada et al., 2002). In addition, the CHOP mRNA includes an upstream inhibitory ORF that is preferentially translated during ER stress (Jousse et al., 2001; Lee et al., 2011). Expression of this protein is very tightly regulated and eventual convergence on CHOP activation signals a likely shift into macroautophagy and/or apoptosis (Gomez-Santos et al., 2005; Kim et al., 2006; Emdad et al., 2011). Thus, PERK has protective functions, especially when first activated, but it can also induce cell death pathways if it is too strongly activated or active for too long. This temporal change in PERK effect has been described in a recent review, identifying PERK as a protein with significant characterization left to be done (Woehlbier and Hetz, 2011).

### PERK AS PROTECTIVE MECHANISM IN MULTIPLE MYELOMA

As previously referenced, Michallet et al. (2011) used RNAi (RNA interference) to individually knock down IRE1, ATF6, and PERK expression. They observed that loss of any one sensor tended to increase the activation read outs of the remaining sensors, confirming crosstalk between the systems. Their specific knockdown of PERK yielded two important findings. First, this single change resulted in an autophagic cell death response, implicating PERK activation as a necessary part of the metabolic shift from plasma cell to myeloma cell. Second, the loss of PERK impeded the apoptotic response. Therefore, PERK activity was implicated in both viability of myeloma cells and in the apoptotic potential of the cells (Michallet et al., 2011). This complex finding may shed light on idea of PERK activity as a potential danger to the cell, which will be discussed in the following section.

PERK status is also a likely factor in myeloma cell response to drug. A report last year noted that myeloma cells demonstrate a baseline degree of UPR that, when inhibited by an HSP90 inhibitor, could result in an apoptotic response (Patterson et al., 2008). This dependence on UPR can therefore be considered an addiction and inhibition of the sensors could constitute a rational drug target. However, a larger body of work has suggested that the downstream effects of PERK activation are identifiable as effectors of cell death in myeloma. It may be that myeloma cells have optimized their ER stress response to survive their unique metabolism as both secretory and rapidly dividing cells. If so, repression of the ER stress response could lead to cell death as surely as stimulation of the same system.

### PERK AS A CELL DEATH EFFECTOR IN MULTIPLE MYELOMA

Activation of PERK has been implicated in a wide variety of cancers as a mediator of response to chemotherapy (Kraus et al., 2008; Lust et al., 2009; Yan et al., 2010; Fribley et al., 2011; Qiao et al., 2012; Sailaja et al., 2013). Most convincingly, small interfering RNA (siRNA) against PERK or dominant negative models can ameliorate chemotherapy-induced death in many types of cancer cells (Lai and Wong, 2008; Yacoub et al., 2008; Kahali et al., 2010; Pan et al., 2012). It is therefore perhaps unsurprising that this effect has also been seen in myeloma cells, which already have baseline ER stress and may not be able to tolerate perturbations to the system. In particular, researchers have been interested in the role of PERK in myeloma cell response to the proteasome inhibitor bortezomib, the most effective myeloma therapy. Obeng et al. (2006) have reported that Bortezomib treatment upregulates PERK activity as measured by ATF4 and downstream CHOP expression. Further, they correlated ER stress to bortezomib response by measuring the retention of immunoglobulin protein accumulating in treated cells. Myeloma cells that retained more of their secretory protein load, one hallmark of ER stress, showed more activation of ER stress markers and more sensitivity to the drug (Obeng et al., 2006).

This pathway has been further probed in myeloma cells by induction of ER stress through inhibition of heat shock proteins, the family of ER chaperones that includes both grp78 and gp96. Most commonly, heat shock protein 90 is targeted experimentally with the drug 17-AAD. A 2007 paper compared 17-AAG and bortezomib effects on myeloma cells and found that both drugs produced upregulation of grp78, gp96, and CHOP, all of which are downstream effects of PERK activation (Davenport et al., 2007). These effects were ultimately joined by an apoptotic response, suggesting that PERK activation culminated in a cell death program (Davenport et al., 2007).

The key component of apoptosis-induction by PERK was investigated to better understand the unfortunate phenomenon of bortezomib resistance in myeloma. Schewe and Aguirre-Ghiso (2009) demonstrated that the phosphorylation of eIF2α is an indispensable aspect of PERK-mediated apoptosis. They studied a bortezomib-resistant subpopulation of myeloma cells and found that resistance could be reversed by inhibition of the eIF2α phosphatase or by competitive inhibition of the phosphatase via overexpression of a mutant phosphorylated eIF2α. In both conditions, cells with experimentally enhanced levels of endogenous phosphorylated eIF2α regained sensitivity to bortezomib (Schewe and Aguirre-Ghiso, 2009).

The global repression of protein translation has far-reaching consequences, even if subsets of mRNAs are selectively processed. For instance, the balance of proteins in the cell quickly changes as proteins with short half-lives are degraded but not replaced. One system affected by such a change is the anti-apoptotic network, comprised of such anti-apoptotic proteins as survivin, Mcl-1, and FLIP (FLICE-like inhibitory protein), all of which are eliminated from the protein pool if not continuously generated (White-Gilbertson et al., 2009). This time-dependent shift in cellular fitness may be another axis on which PERK activation is titrated, so that short-term activation is beneficial while long-term activation is ultimately detrimental to the cell.

## CONCLUSION

The integrated ER stress response is composed of all three sensor systems and their interplay determines the overall cellular strategy and the outcome of stress. Myeloma cells may harbor an Achilles heel in their baseline metabolism, as shown by varied treatments which induce death by either inhibiting or exacerbating the ER stress response. This metabolic addiction to pathways that prevent UPR-induced death program may be a key to myeloma treatment, and deserves more focused attention. One example of such effort is the possibility of PERK inhibitors as cancer therapeutics (Bi et al., 2005; Hart et al., 2012). It is also possible that a unique adaptive UPR program is adopted by individual myeloma patients, having diseases with different vulnerabilities. An individualized strategy with an array of tools to inhibit or push ER stress may be needed to match therapeutic response to this adaptive disease.

In summary, the UPR mechanism can be exploited for the treatment of MM (**Figure 1**). The shift from naïve B cell to plasma cell already involves the activation of two of the three ER stress sensors and their downstream signaling partners (**Figures 1A,B**). These cells engage IRE1 and ATF6 in order to cope with regulated antibody production, although PERK is inhibited. However, upon transformation, myeloma cells require the further support of PERK, allowing transcription of the ATF4 targets that ameliorate oxidative stress (**Figure 1C**). This is a potentially risky strategy for the cell, because ATF4 and ATF6 can cooperate to transcribe the pro-apoptotic CHOP (**Figure 1D**). Thus, if chemotherapeutic interventions can tip the balance of the ER stress response into supporting programmed cell death, they would be leveraging the intrinsic weakness of the disease, to have a desired treatment outcome for MM.

## Conflict of Interest Statement

The authors declare that the research was conducted in the absence of any commercial or financial relationships that could be construed as a potential conflict of interest.
